# Frameless intraputaminal delivery of gene therapy with eladocagene exuparvovec in patients with aromatic L-amino acid decarboxylase deficiency: safe and efficient results

**DOI:** 10.1007/s00381-025-07020-y

**Published:** 2025-11-10

**Authors:** Roni Mai, Dmitriy Reshchikov, Vladimir Popov, Sergey Gorelikov, Ekaterina Zakharova, Svetlana Mikhaylova

**Affiliations:** 1 Department of Pediatric Neurosurgery, Russian Children’s Clinical Hospital, Pirogov Russian National Research Medical University, Moscow, Russia; 2 Department of Pediatric Surgery, Moscow Regional Scientific Research Clinical Institute M.F. Vladimirsky, Moscow, Russia; 3 Federal State Budgetary Scientific Institution “Academician N.P. Bochkov Research Centre for Medical Genetics”, Moscow, Russia

**Keywords:** AADC deficiency, Frameless stereotaxis, Gene therapy, Pediatric neurosurgery, Oculogyric crises, Neurotransmitter disorders

## Abstract

**Purpose:**

We present two clinical cases of frameless, neuronavigated gene therapy with eladocagene exuparvovec for aromatic L-amino acid decarboxylase (AADC) deficiency in pediatric patients, detailing the targeted bilateral microdose delivery of viral vectors into the putamen and highlighting the feasibility and challenges of this approach in managing a rare neurometabolic disorder.

**Methods:**

Two patients with a confirmed diagnosis of AADCD underwent frameless stereotactic gene therapy. High-resolution 3 T MRI-guided trajectories were planned for the targeted bilateral putaminal infusion of 0.32 ml of eladocagene exuparvovec (AAV2-hAADC). The agent was delivered via a SmartFlow Neuro Ventricular Cannula in a “Z-pattern,” retracting the cannula 2 mm every 9 min to achieve controlled microdosing.

**Results:**

Accurate frameless drug delivery was achieved in a shorter time than frame-based approaches, with no intraoperative or postoperative complications. One patient showed a small post-ischemic cyst on the 1-month follow-up MRI, without any neurological deficits. Over 2 months, both patients demonstrated reduced oculogyric crises, diminished hyperkinesis, and improved head control, with no significant adverse events.

**Conclusion:**

Frameless, neuronavigated gene therapy for AADC deficiency proved both feasible and safe, with early clinical improvements observed in motor function and symptom control. This technique offers a promising alternative to frame-based methods and expands treatment options for this rare neurometabolic disorder.

## Relevance

Aromatic L-amino acid decarboxylase (AADC) is involved in the synthesis of serotonin and dopamine, with dopamine serving as a precursor to epinephrine and norepinephrine. AADC deficiency is a rare autosomal recessive neurometabolic disorder that leads to a deficiency of serotonin and dopamine. This dopamine deficiency results in dysfunction of the nigrostriatal dopaminergic pathway. Clinically, reduced activity in this pathway is characterized by dystonia, tardive dyskinesia, ataxia, and parkinsonism [1–6]. Also the most common signs include developmental delay and dysautonomia (abnormal sweating, bradycardia, etc.) [5].

Since the disease was first described in the 1990 s, only rare case reports have been published, predominantly involving patients from Asia. The incidence of this disease remains unknown [5, 6]. Symptoms typically begin within the first months of life and are characterized by motor disturbances; however, cases with a mild disease course have also been reported.

Treatment of this disease is challenging due to its extremely rare occurrence and the lack of extensive research. Only expert opinions from a few centers worldwide are available. Some patients have responded well to levodopa treatment; others have been prescribed dopamine agonist replacement therapy [5, 6]. However, most patients have been resistant to therapy. To address the treatment of disorders like aromatic L-amino acid decarboxylase deficiency (AADCD), an international group for the study of neurotransmitter disorders was established [6].

In 2017, Wassenberg et al. published guidelines on the diagnosis and medical treatment of AADCD [6]. Patients with mild presentations of the disease respond to medical therapy; however, treatment options remain limited, and many patients succumb to the condition in early childhood. Surgical treatment has been introduced relatively recently and is currently available in only a few countries. Surgical treatment includes the viral vector (adeno-associated virus type 2) gen delivery to the bilateral putamen [7].

To date, only a limited number of procedures involving the stereotactic delivery of viral preparations into the putamen have been reported, all performed using frame-based stereotaxy with intraoperative MRI/CT guidance [1, 3]. Here, we describe two cases of surgical treatment for AADCD. These cases involved the targeted, bilateral microdose delivery of viral vectors carrying gene therapy directly into the putamen. The procedures were performed using frameless Brainlab neuronavigation combined with VarioGuide-assisted techniques. Both cases were conducted at the Russian Children’s Clinical Hospital in Moscow, Russia.

## Materials and methods

### Patient selection

Eladocagene exuparvovec (Upstaza®) is a gene therapy product that contains an adeno-associated virus serotype 2 (AAV2)-mediated human aromatic L-amino acid decarboxylase (AADC) gene (AAV2-hAADC). In the Russian Federation, the use of unregistered medicinal products is allowed for life-threatening conditions in accordance with Part 3, Article 37 of Federal Law No. 61-FZ “On the Circulation of Medicines.” For children with severe and orphan diseases, such drugs are procured using funds from the “Circle of Kindness” foundation, based on Federal Law No. 48-FZ of March 26, 2021. Patients were selected based on their clinical presentation and confirmed genetic testing by the Genetics Department of the Russian Children’s Clinical Hospital, Pirogov Russian National Research Medical University (Moscow, Russia), and were subsequently recommended to undergo surgical treatment with this gene therapy product.

### Patient presentation

Patient 1 (P1) (Fig. 2) is a 6-year-old with a pathogenic DDC variant (c.1073G > A/p.Arg358His). He presents with severely delayed motor skills (inability to hold his head up, sit, crawl, or walk), delayed speech development, excessive sweating, and choking when swallowing (Table 1) Prior to gene therapy, his medical treatment included the following: Selegiline (MAO-B inhibitor): 0.000164 g q12hr. Pramipexole (dopamine agonist), 0.25 mg q6.hr; trihexyphenidyl (anticholinergic), 7 mg q6hr, alternating with 8 mg q6.hr; pyridoxine hydrochloride (vitamin B6), 100 mg q12.hr; Folic acid (vitamin B9), 1 mg q12.hr; melatonin (sleep regulator), 3 mg once daily at bedtime.

**Table 1:** Clinical signs of AADCD in our patients

Patient	P1	P2
1. Hypotonia	++	+++
2. Developmental delay	++	+++
3. Movement disorders		
a. Dystonia	+	+
c. Oculogyric crisis	+	+++
e. Tardive dyskinesia		+
g. Ataxia	++	+
i. Parkinsonism	-	+
4. Dysautonomia		
a. Abnormal sweating	+++	+++
c. Excessive drooling	+	+++
e. Hypotension	+ \-	+
g. Bradycardia	-	++
i. Temperature instability	-	++

*P1* patient 1, *P2* patient 2 (+, mild; ++, moderate; +++, severe)

Patient 2 (P2) (Fig. 3) is a 7-year-old with a pathogenic variant in the DDC gene (c.818C > A/p.A273D). He presents with dystonic attacks, constant oculogyric crises, hypersalivation, sleep disturbances, and cachexia (weight 10.3 kg) (Table 1). Medical treatment prior to gene therapy included the following: selegiline (MAO-B inhibitor), 0.3125 mg every 12 h; pramipexole (dopamine agonist), 0.1875 mg every 8 h; trihexyphenidyl (anticholinergic), 0.5 mg every q12hr; pyridoxal phosphate (vitamin B6), 50 mg q12hr; melatonin, 1.5 mg at bedtime; diazepam, administered as needed for clinical indications.

### Methods

The procedure was performed using the frameless stereotactic Brainlab system, including the Brainlab planning station and the VarioGuide navigation system. Imaging was conducted with a 3-Tesla MRI scanner capable of acquiring high-resolution images in 3D TOF (time-of-flight) and T2 CUBE modes with a slice thickness of 0.6 mm. Following the MRI scan, the brain images were uploaded to the planning station. Based on recommendations from clinical studies [8], two trajectories were planned for each putamen: one targeting the anterior-middle third and the other the posterior-middle third.

The SmartFlow Neuro Ventricular Cannula (16ga, 0.008″ I.D. × 4 ft with 18-mm tip, catalog no. NGS-NC-01, ClearPoint Neuro) was designed for human application and was used to deliver the therapeutic agent into the putamen. The gene product was administered in a single procedure by convection-enhanced infusion (CED) at 0.003 mL/min (0.18 mL/h) for 27 min per target (≈0.081 mL per target), across four targets (two per putamen), for a total infused volume ≈0.324 mL (reported as 0.32 mL; 0.16 mL per putamen) and a total patient dose of Upstaza® 1.8 × 10^11^ vector genomes. Each trajectory we used a 9 + 9 + 9-min infusion scheme, followed by a 5-min pause after the final (third) 9-min infusion, with the cannula left in place to allow the pressurized column within the cannula to discharge into tissue and minimize reflux. Because delivery relies on a pressure gradient, the pump pressure-alarm threshold was set > 16 psi (~ 825 mmHg). 

### Navigation and planning

**Fig. 1:**
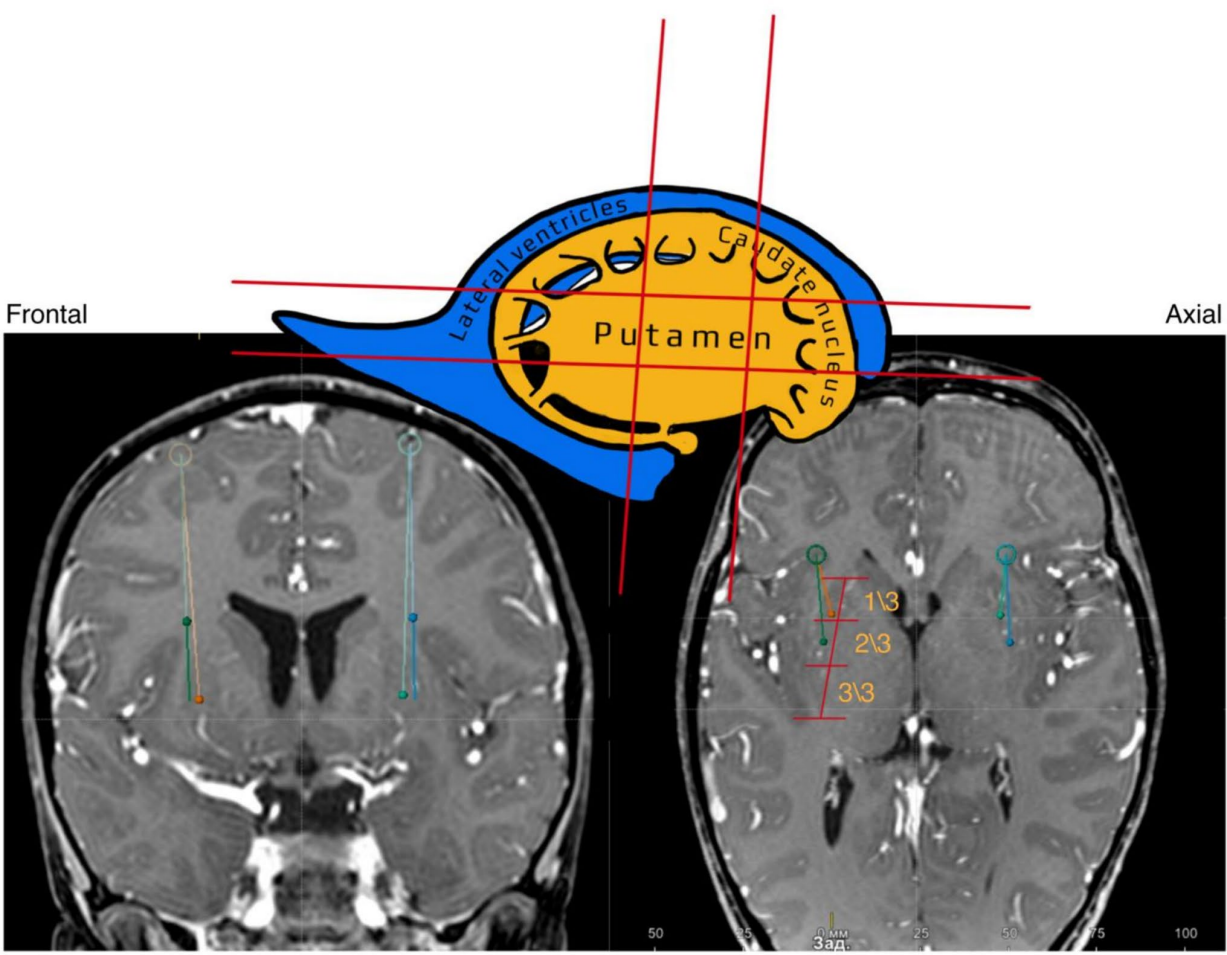
A schematic representation of the putamen. Red lines indicate the points used to subdivide the putamen for planning drug administration. On axial slices, the putamen is divided into anterior and posterior thirds; on frontal slices, it is divided into upper, middle, and lower levels. The images from the Brainlab planning station illustrate trajectories tailored to the patient’s unique anatomical features. In the axial view, a perforating lenticulostriate artery is located at the posterior planning point, necessitating an anterior shift of the posterior trajectory to preserve an 8-mm safe distance between the anterior and posterior infusion trajectories

The trajectory planning ensures that the drug is distributed at the highest possible dose within the putamen while minimizing the risk of tissue ischemia from volume-induced compression. According to current recommendations, the putamen is divided into three segments in the anteroposterior (axial) dimension and three levels in the superoinferior (frontal) dimension. In addition, it is subdivided into right and left halves in the axial plane. The overall segmentation scheme and the safe injection zone within the putamen are illustrated in (Fig. 1). The distance between the anteroposterior trajectories should be at least 8 mm, and the projection of the entry point is positioned on the cortical surface.

### Surgical procedure

We place the stereotactic entry points anterior to the coronal suture, thereby minimizing the risk of injury to large bridging veins. For optimal access to two trajectories on each side, a single linear skin incision is made at the projection of the entry points. Two large-diameter burr holes are created to allow access to both trajectories from a single burr hole. The dura mater is opened to verify cortical landmarks and confirm the depth of cannula insertion.

**Fig. 2:**
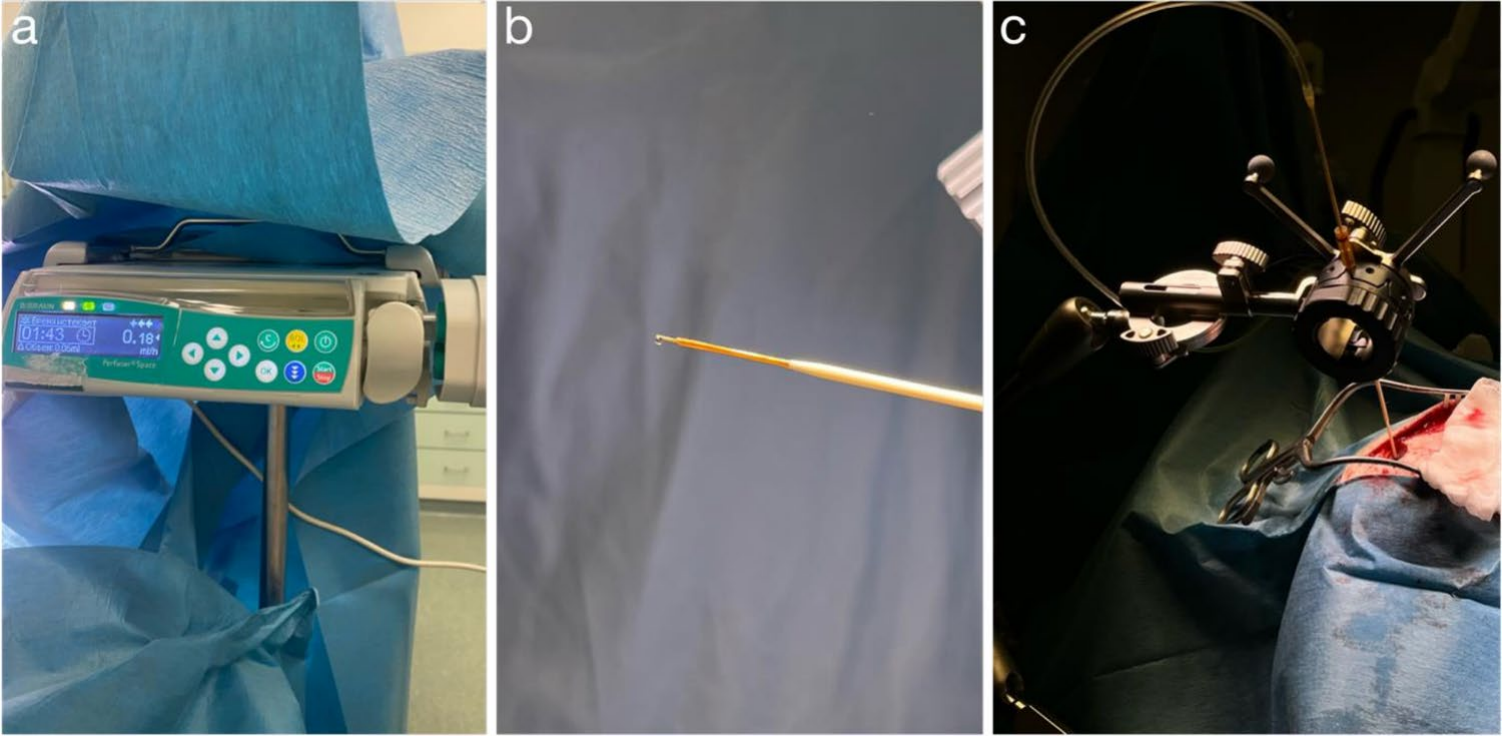
**a** Infusion pump with loaded syringe (flow rate 0.18 mL/h). **b** First droplet of product at the cannula tip. **c** Cannula seated in the VarioGuide and advanced to the target through a burr hole

The cannula was connected to the product syringe, the syringe was loaded into the infusion pump, and all parameters were set as described above. The line was then primed at up to 0.003 mL/min (0.18 mL/h) (Fig. 2a) until a single droplet appeared at the cannula tip, after which the pump was stopped (Fig. 2b). The infusion follows a Z-pattern: first, the anterior left trajectory, followed by the anterior right, then the posterior left, and finally the posterior right. The cannula itself is not pre-graduated; therefore, upon reaching the final target point, a zero mark was manually designated on the cannula using a sterile marker. Subsequently, the cannula was retracted by 2 mm every 9 min, with a new zero mark applied at each step.

## Results

The performed surgeries and postoperative evaluations confirmed accurate drug delivery to the target, with a deviation from the initial planned entry points of ± 1 mm. This deviation is considered acceptable, as the center of the putamen was used as the reference point. The operative time from incision to closure was 4 h. The delivered volume was consistent with clinical recommendations (0.32 mL total, with 0.16 mL administered to each putamen) and was confirmed by data from the B.Braun infusion pump at the end of each trajectory.

**Fig. 3:**
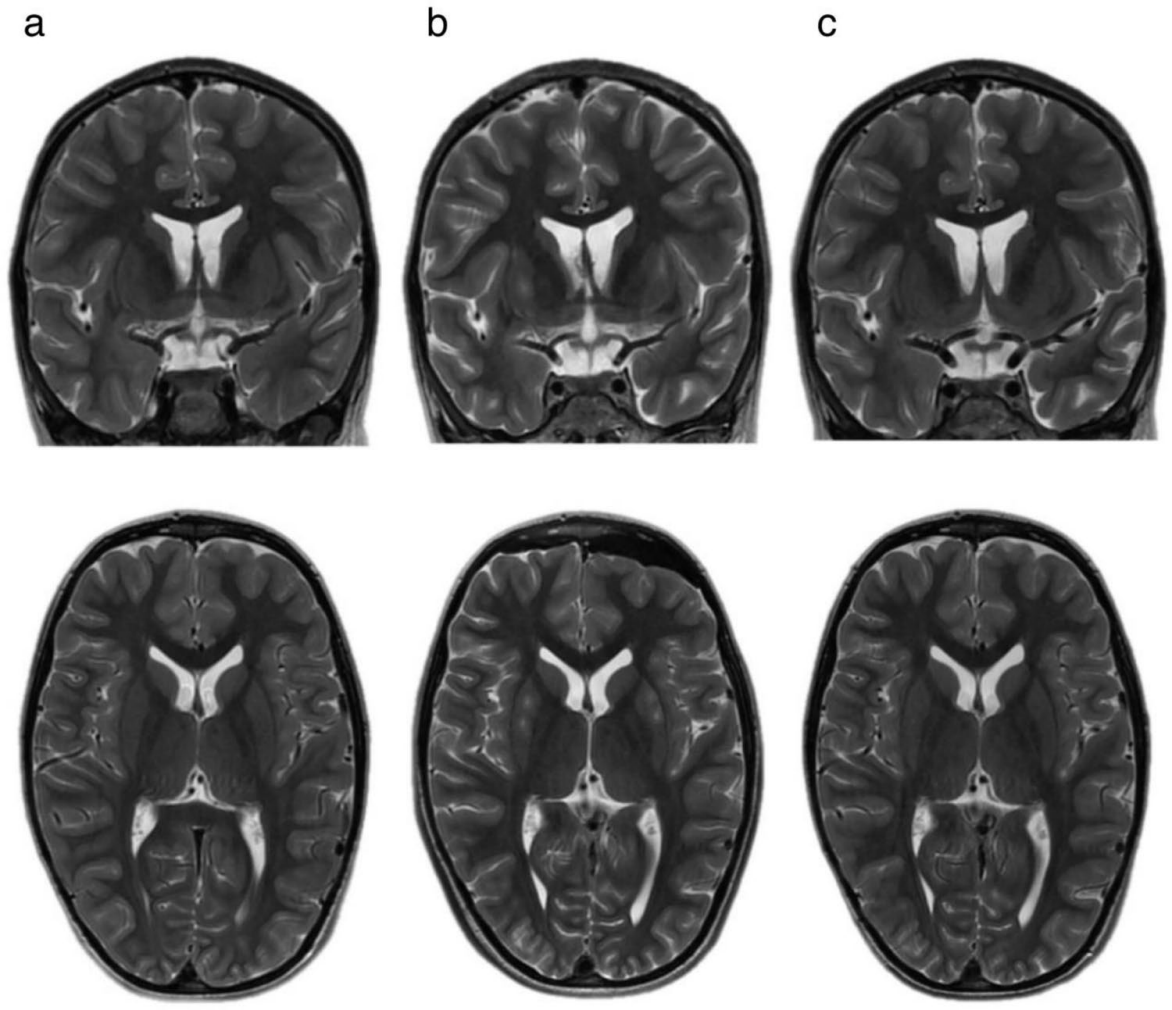
MRI scans of P1: A patient with preserved brain volume. **a** Preoperative T2-weighted images in frontal and axial views. **b** Postoperative T2-weighted images showing post-puncture trajectories. **c** T2-weighted images on postoperative day 30, demonstrating the absence of abnormalities or post-puncture tracts

**Fig. 4:**
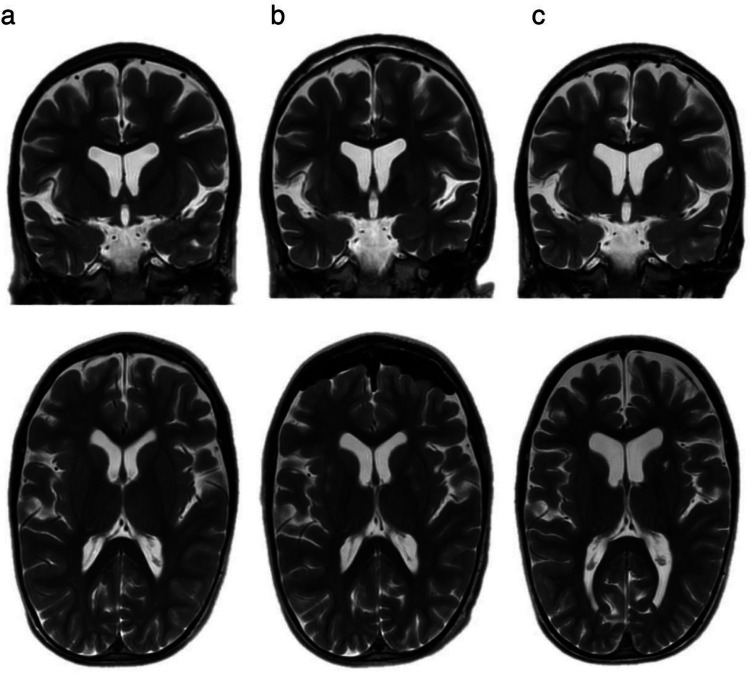
MRI scans of P2: A patient with cerebral atrophy. **a** Preoperative T2-weighted images in frontal and axial views. **b** Postoperative T2-weighted images in frontal and axial views showing post-puncture trajectories, with no signs of hemorrhagic stroke. **c** T2-weighted images on postoperative day 30 in frontal and axial views demonstrating a post-hemorrhagic infarction in the anterior-superior regions of the left putamen

Immediate postoperative MRI scans (Fig. 3b) (Fig. 4b) revealed no evidence of intraoperative bleeding or other procedure-related complications. No postoperative neurological deficits were observed compared to the patient’s preoperative baseline. Both patients were discharged on the 7th postoperative day and transferred to the Genetics Department for further observation.

Follow-up MRI at 1 month postoperatively (Fig. 3c) (Fig. 4c) revealed a small post-ischemic cyst in the anterosuperior region of the putamen in Patient 2, likely due to a microhemorrhage or a post-trajectorial effect from the cannula with no new neurological deficits observed. The postoperative clinical progression of patients is summarized in Table 2.

**Table 2:** Summary of the changes in patient symptoms at postoperative follow-up intervals, illustrating the overall trajectory of clinical improvement

Patient	First week	Second week	30 days after surgery	60 days after surgery
P1	Developmental delay and hyperkinesis, with oxygen saturation decreasing to 92%. OCG stopped entirely	Developmental delay persisted, and hyperkinesis increased	Developmental delay was still present, though hyperkinesis slightly decreased. The patient was able to hold their head upright and roll over	Hyperkinesis had further diminished. Head control improved, rolling over was quicker, and the patient began to form individual words
P2	Dystonic attacks, diarrhea, sleep disturbances, crying, irritability, feeding difficulties, respiratory failure (oxygen saturation at 84%), somnolence, weight loss, and increased saliva production	The patient continued to have dystonic attacks, facial hyperkinesis, diarrhea, sleep disturbances, crying, feeding difficulties, and increased saliva production. Weight loss persisted, abnormal breath sounds were noted, serum potassium was low, OCG appeared, and the patient demonstrated some head control	The patient continued to exhibit dystonic attacks, sialorrhea, and hyperkinesis in the facial muscles and limbs, with occasional OCG. Oxygen saturation was at 94% while breathing room air (without an oxygen mask)	No dystonic attacks or sialorrhea were reported. Hyperkinesis persisted in the facial muscles and limbs, with rare OCG. The patient began to hold their head upright and roll onto one side from a supine position

*P1* patient 1, *P2* patient 2; *OCG*, oculogyric crises

## Discussion

According to a systematic review and meta-analysis by Kesserwan et al., there is no statistically significant difference in diagnostic yield between frame-based and frameless stereotactic brain biopsies [9]. In a randomized trial, Bradac et al. reported mean target deviation of 2.65 ± 1.12 mm (frame) versus 2.90 ± 1.26 mm (VarioGuide), *p* = 0.456, indicating equivalent precision [10]. Moreover, frameless stereotactic targeting of deep nuclei in NHPs has been shown to be feasible, accurate, and safe, underscoring the practicality of a frameless parenchymal approach [11]. Consistent with these data, we selected a frameless VarioGuide workflow because it provides frame-comparable accuracy with shorter setup and anesthesia. For putaminal CED, safety depends primarily on preoperative trajectory planning (avoiding lenticulostriate arteries/internal capsule) and controlled infusion parameters rather than sub-millimetric differences in navigation metrics. Operationally, the frameless approach obviates frame application, scanning and transport/re-draping, reducing anesthesia time by ~ 30–90 min; in our pediatric AADCD series, bilateral intraputaminal microdose delivery was completed in ~ 3.55 h from incision to closure—shorter than the 6–8 h durations (per information supplied by the manufacturer). Minimizing intraoperative time directly supports Enhanced Recovery After Surgery pathways. However, there are certain challenges associated with large burr hole craniotomies.

The first challenge is cerebral cortical atrophy in these children [6]. Upon craniotomy, the release of cerebrospinal fluid (CSF) due to positive pressure and the risk of pneumocephalus can lead to brain shift relative to the preoperatively planned MRI-based trajectories. Predicting the direction and magnitude of this shift is difficult. A gap of approximately 1–3 mm can form between the dura mater and the cortex; at this point, it is essential to assess this distance and adjust the cannula insertion depth accordingly. However, as demonstrated in Patient 2, this was not a significant concern, given that the trajectories were initially planned from the center of the putamen, permitting a 1–2 mm deviation in either direction without compromising accuracy. Nonetheless, we cannot extrapolate these findings to cases where the putamen is substantially smaller than in our patient cohort, as we have not yet encountered such cases in our practice. All patients underwent immediate postoperative brain MRI to verify infusion accuracy.

The second challenge is postoperative CSF leakage. CSF leakage can be minimized by performing a linear incision of the dura and achieving a watertight closure, with additional sealing using TachoComb to reinforce the primary suture. We backfilled the burr hole with autologous bone dust and applied an additional layer of TachoComb over the bone. No postoperative cerebrospinal fluid collections were observed in our series.

Preoperative planning is crucial, and image fusion helps avoid trajectories involving medium- and large-caliber blood vessels. If a perforating lenticulostriate artery supplying the putamen is located at the planned target, it is acceptable to shift the trajectory anteriorly or posteriorly to ensure patient safety, even though the standard recommendation is to target the center of the putamen. It is crucial to remain within the anatomical boundaries of the putamen. In one patient, MRI performed 1 month postoperatively revealed a cystic cavity in the upper portion of the putamen along the injection trajectory. In a long-term follow-up study, Chun-Hwei Tai et al. demonstrated the safety of putaminal viral vector delivery, and the operative tracts had no clinical significance even 7 years after the procedure [4].

Compared with CSF administration, direct parenchymal CED achieves higher drug concentrations at the putaminal target with more predictable tissue coverage, whereas CSF dilution and rapid clearance limit penetration into deep nuclei. Recent iMRI-guided transaxial intraputaminal infusions (25–50 µL at 2.5 µL/min, 2-mm spacing, 10-min wait before withdrawal) demonstrated reliable on-target delivery, limited backflow, and no persistent deficits, while reducing the number of required tracks across the putamen [12]. Consistent with these data, we infused at a low rate of 0.003 mL/min (3 µL/min), consistent with preclinical practice and not associated with putaminal injury. The dose was divided across three sequential depths (9 + 9 + 9 min with 2-mm step-ups), followed by a 5-min pause after the final (third) 9-min infusion—a scheme that lowers focal tissue pressure, limits reflux, and helps protect adjacent nuclei.

We observed an early positive neurological outcome in both patients. Although Patient 2 had a trajectory deviation resulting in infusion along the putaminal margin on one side, this did not affect the overall result. Notably, our patient with a more severe phenotype improved more rapidly than the one with a moderate form. By the second postoperative week, the patient could hold their head upright independently—a function that was previously absent. This rapid clinical improvement is notable, especially in the context of severe AADC deficiency, which is generally associated with poor or significantly delayed therapeutic response [5, 8].

Among patients receiving pharmacological treatment—regardless of disease severity—vitamin B6 resulted in a 38% positive response rate, dopamine agonists 55%, and MAO inhibitors 56% [5]. However, the response was generally limited to patients with mild or moderate phenotypes, with little or no effect observed in most severely affected individuals. In contrast, our experience with gene therapy demonstrated a clear and early therapeutic effect in a patient with a severe phenotype. Nevertheless, additional research and long-term follow-up are essential to determine the sustained efficacy of gene therapy and its potential to benefit a broader spectrum of patients, particularly those with severe phenotypes who have historically shown limited response to conventional pharmacological treatments.

## Conclusion

Frameless stereotactic intraputaminal infusion is user-friendly, safe, and time-efficient compared to frame-based stereotactic intraoperative MRI-guided infusion. In patients with AADC deficiency, it reduces anesthesia time and minimizes the risks associated with prolonged anesthesia. A positive outcome from gene therapy is already evident within a short follow-up period, and the more severe form appears to respond sooner than the moderate form.

## Limitations

Limitations include the small sample size (*n* = 2) and short follow-up period.

## Data Availability

No datasets were generated or analysed during the current study.
